# Hsa_circ_0003611 hinders the transformation of mesenchymal stem cells into osteosarcoma cells through suppressing MYC by IGF2BP3 via m^6^A modification

**DOI:** 10.1186/s40659-025-00659-6

**Published:** 2025-11-29

**Authors:** Fei Zhang, Fei Cheng, Zhiyong He, Chengyi Zhao

**Affiliations:** 1 First Department of Orthopaedics, Zhongshan City People’s Hospital, Zhongshan, 528403 Guangdong China; 2 Second Ward of Cardiovascular Medicine, The Affiliated Dongguan Songshan Lake Central Hospital, Guangdong Medical University, Dongguan, 523326 Guangdong China; 3 Second Department of Orthopaedics, Zhongshan City People’s Hospital, Zhongshan, 528403 Guangdong China

**Keywords:** Osteosarcoma, Mesenchymal stem cell, Transformation, Hsa_circ_0003611, MYC, m^6^A modification

## Abstract

**Background:**

Osteosarcoma (OS) is the most common non-hematogenous primary malignancy in the bone. Due to several origins of OS, 30–40% OS patients would experience recurrence and metastasis, with a 5-year survival rate of 20–30%. Mesenchymal stem cells (MSCs) transform into OS cells during the differentiation into osteoblasts, and circular RNA (circRNA) hsa_circ_0003611 might contribute to the differentiation of MSCs into osteoblasts. However, the role of hsa_circ_0003611 in the transformation of MSCs into OS cells is largely unknown. This study aims to investigate whether hsa_circ_0003611 tunes the transformation of MSCs into OS cells.

**Methods:**

Here, human bone marrow mesenchymal stem cells (hBMSCs) with hsa_circ_0003611 stably silenced was constructed. Moreover, protein-RNA interaction was detected by RNA immunoprecipitation (RIP), and N^6^-methyladenosine (m^6^A) modification of hsa_circ_0003611 was determined using methylated RNA immunoprecipitation (MeRIP).

**Results:**

The present study reveals that hsa_circ_0003611 level is almost absent in OS cells compared to that in osteoblasts and MSCs. Moreover, hsa_circ_0003611 silence enhances the transformation of MSCs into OS cells in vitro and triggered tumorigenicity of MSCs for OS in vivo. Mechanistically, silence of hsa_circ_0003611 promotes the transformation of MSCs into OS cells by activating MYC proto-oncogene, bHLH transcription factor (MYC) via insulin like growth factor 2 mRNA binding protein 3 (IGF2BP3). Moreover, hsa_circ_0003611 silence improves MYC mRNA stability by facilitating the association between IGF2BP3 and MYC mRNA in MSCs. Furthermore, m^6^A modification disrupts the association between hsa_circ_0003611 and IGF2BP3 to enhance the association between IGF2BP3 and MYC mRNA in MSCs.

**Conclusion:**

In summary, these findings highlight the role of hsa_circ_0003611 in the transformation of MSCs into OS cells and provide novel targets and strategies for OS treatment.

**Supplementary Information:**

The online version contains supplementary material available at 10.1186/s40659-025-00659-6.

## Introduction

Osteosarcoma (OS) is the most common non-hematogenous primary malignancy of the bone, accounting for 33.3% cases of primary malignancy in bone and causing a high rate of mortality [1, 2]. OS mainly affects young and elderly individuals [3]. Combining surgery with radiotherapy, chemotherapy, immunotherapy and targeted drugs has immensely improved the 5-year survival rate of OS patients. However, 30–40% patients would experience recurrence and metastasis, with a 5-year survival rate of 20–30% [4]. The main reason for this could be returned to several origins of OS [5]. Therefore, the mechanisms regulating OS origin must be elucidated to develop effective strategies for OS treatment.

Mesenchymal stem cells (MSCs) transform into OS cells during the differentiation into osteoblasts, which is validated by increased OS markers including T-box transcription factor 3 (Tbx3) in MSCs [5–7]. However, the hub genes and mechanism regulating the transformation of MSCs into OS cells remains unclear. Growing evidence have indicated the critical role of circular RNAs (circRNAs) in OS. For instance, circRNA PVT1 (circPVT1) is correlated with poor prognosis of OS patients and facilitates metastasis of OS cells by regulating microRNA (miRNA) miR-526b [8]. Similarly, circRNA from transcriptional adaptor 2 A (circTADA2A) sponges miR-203a-3p as a competing endogenous RNA (ceRNA) to promote the progression and metastasis of OS [9]. By contrast, hsa_circ_0002052 suppresses OS progression through miR-1205/ APC regulator of WNT signaling pathway 2 (APC2) pathway [10]. We found that hsa_circ_0003611 is downregulated in OS tissues by screening circRNA expression profiles in OS tissues (GSE96964) obtained from Gene Expression Omnibus (GEO) DataSets, and it might contribute to the differentiation of MSCs into osteoblasts. However, the role of hsa_circ_0003611 in the transformation of MSCs into OS cells is largely unknown.

It has been reported that overexpression of MYC proto-oncogene, bHLH transcription factor (MYC) would induce the transformation of MSCs into OS cells [11, 12]. However, the mechanism has not been explored. Numerous studies have revealed the regulatory effects of circRNAs on MYC in various cancers [13–16], yet the role of hsa_circ_0003611 in MYC activation during the transformation of MSCs into OS cells has not been reported.

A recent study has indicated that insulin like growth factor 2 mRNA binding protein 3 (IGF2BP3) facilitates OS malignancy by improving ubiquitination factor E4A (UBE4A) mRNA stability [17]. Moreover, IGF2BP3 also enhances MYC mRNA stability in HEK293T cells [18]. Nevertheless, the effect of IGF2BP3 on MYC mRNA in OS remains unclear. Additionally, circRNAs contribute to IGF2BP3-mediated mRNA stability. In triple-negative breast cancer, circBRAF promotes the association between IGF2BP3 and target mRNAs to improve the stability of target mRNAs [19]. However, the role of hsa_circ_0003611 in IGF2BP3-dependent mRNA stability has not been reported. Therefore, whether hsa_circ_0003611 tunes MYC mRNA stability by IGF2BP3 during the transformation of MSCs into OS cells needs to be investigated.

N^6^-methyladenosine (m^6^A) is the most abundant post-transcriptional RNA modification in eukaryote [20]. MSCs transform into OS cells during the differentiation into osteoblasts [5–7], and m^6^A modification triggers the differentiation of MSCs into osteoblasts [21]. Moreover, m^6^A modification facilitates circBRAF-IGF2BP3 interaction to increase IG2BP3 target mRNA stability in breast cancer [19]. However, whether m^6^A modification contributes to the transformation of MSCs into OS cells by regulating MYC mRNA stability via modulating hsa_circ_0003611-IGF2BP3 interaction has not been elucidated.

Thus, the current study aims to investigate whether hsa_circ_0003611 tunes the transformation of MSCs into OS cells through modulating MYC by IGF2BP3 via m^6^A modification.

## Materials and methods

### Data collection and analysis

Gene expression profiles of circRNAs (GSE96964 and GSE140256) were obtained from the GEO DataSets of National Center for Biotechnology Information (NCBI).

### Cell culture

Human bone marrow mesenchymal stem cells (hBMSCs), human osteoblast cell line hFOB1.19 cells, human OS cell line 143B, HOS and MG63 cells were obtained from The Cell Bank at the Chinese Academy of Sciences (Shanghai, China). hBMSCs were cultured with human bone marrow mesenchymal stem cell complete medium (#HUXMA-9001c, Cyagen, Suzhou, Jiangsu, China) containing 10% fetal bovine serum (FBS), 100 U/mL penicillin and 100 µg/mL streptomycin. hFOB1.19 cells were grown in DMEM/F12 medium (#SH30023.01B, HyClone, Logan, UT, USA) consisting equal FBS, penicillin and streptomycin to hBMSCs. Moreover, 143B, HOS and MG63 cells were cultured in DMEM medium (#SH30261.01, HyClone) supplemented with equal FBS, penicillin and streptomycin mentioned above. All cells were incubated at 37 °C in a humidified atmosphere of 5% CO_2_.

### RNase R treatment

To characterize hsa_circ_0003611, 4 µg total RNA extracted from hBMSCs by TRIzol (#15596026CN, Invitrogen, Carlsbad, CA, USA) was incubated with 4 U/µg RNase R (#R0300, Geneseed Technologies, Guangzhou, Guangdong, China) for 10 min at 37 °C.

### Quantitative reverse transcription-PCR (qRT-PCR)

Total RNA was extracted from hBMSCs, hFOB1.19, 143B, HOS and MG63 cells using TRIzol (#15596026CN, Invitrogen). Then first-strand cDNA was made utilizing PrimeScript II 1st Strand cDNA Synthesis Kit (#6210A, Takara Biotechnology, Daliang, Liaoning, China). Next, qRT-PCR was performed by TB Green^®^ Premix Ex Taq™ (Tli RNaseH Plus) (#RR420A, Takara Biotechnology) using 10 μm of each primer listed in Table 1 and 150ng cDNA. Results of the target RNAs were normalized to that of GAPDH and presented as 2^−△△Ct^ values relative to those of control samples.

**Table 1:** Sequences of primers used for qRT-PCR

Genes		Sequences (5’-3’)
hsa_circ_0003611	Forward	CGCCAGAGGACTATGAGAATG
	Reverse	GATAGGTGGATGGGGAGCTT
MYC	Forward	CATACATCCTGTCCGTCCAAG
	Reverse	GAGTTCCGTAGCTGTTCAAGT
MINA53	Forward	ACAGTACTGCCGGATCAAGATC
	Reverse	GGAAGCGAAGTCCATGAAACTC
DKC1	Forward	TCATCTCTACCTGCGACCATG
	Reverse	AGAAGGCCCTGCTTGATCATC
IGF2BP3	Forward	GTCAAGTGCAGAAGTTGTTGTC
	Reverse	CTGTTGTTGGTGCTGCTTTAC
FTO	Forward	GGATGAAGGAGAGACAGATGAAG
	Reverse	GTCAGCTAAACCTACATCCCTGA
GAPDH	Forward	AACGGATTTGGTCGTATTGGG
	Reverse	CCTGGAAGATGGTGATGGGAT

### Establishing a stable cell line

In this study, the lentivirus interference vector pLVX-shRNA1 was utilized to establish hBMSCs with hsa_circ_0003611 stably silenced. To target hsa_circ_0003611, a specific sequence of shRNA was created based on hsa_circ_0003611 siRNA. Then two single DNA strands of shRNA targeting hsa_circ_0003611 were cloned into the lentivirus interference vector pLVX-shRNA1 to produce the lentivirus shRNA interference vectors of hsa_circ_0003611. Next, lentivirus shRNA interference vectors of hsa_circ_0003611 were co-transfected with lentivirus packaging helper plasmids pLP1 and pLP2 into 293 FT cells using Lipofectamine 2000 (#11668019, Invitrogen). After collecting and concentrating lentivirus stock solution, the lentivirus titer was identified. Subsequently, hBMSCs were incubated with conditioned medium containing lentivirus supplemented with 5 µg/mL polybrene for 48 h. Transfected cells were selected with puromycin to generate stable hBMSCs with hsa_circ_0003611 silenced (sh-hsa_circ_0003611) and verified by qRT-PCR. The primers used for qRT-PCR were present in Table 1.

### Hematoxylin and Eosin (H&E) staining

For cells, hBMSCs and HOS cells were collected and fixed overnight with 4% paraformaldehyde (PFA). For xenograft tumors derived from nude mice, mice were anesthetized with pentobarbital and then euthanized by CO_2_. Next, xenograft tumors were collected and fixed overnight with 4% PFA and then embedded in paraffin wax to section into 5 μm thickness. Subsequently, H&E staining was performed using Hematoxylin-Eosin/HE Staining Kit (#G1120, Solarbio Biotechnology, Beijing, China) according to the instructions of manufacturer. After staining, the images of cells and xenograft tumors were captured by microscope (Eclipse Ci-L, Nikon. Tokyo, Japan).

### Western blot (WB)

Proteins were firstly extracted from cells or tumor tissues by RIPA lysis buffer (#P0013B, Beyotime, Shanghai, China). Then 30 µg proteins were loaded into SDS-polyacrylamide gel containing 10% polyacrylamide and separated by electrophoresis. Electrophoresis was performed for 50 min using 200 V at 25 °C. Next, proteins were transferred onto the PVDF membrane (#IPVH00010, Millipore, Bedford, MA, USA) followed by blocking membranes using 5% non-fat milk for 2 h at 25 °C. Subsequently, membranes were incubated with primary antibodies overnight at 4 °C. The next day membranes were incubated with secondary antibody for 1 h at 25 °C. Then the signals of target proteins were visualized by BeyoECL Moon (#P0018F, Beyotime). The primary antibodies used for WB included MYC antibody (1:1000, #67447-1-Ig, Proteintech, Wuhan, Hubei, China), Tbx3 antibody (1:500, # ab89220, Abcam, Cambridge, UK), and GAPDH antibody (1:10000, #KC-5G5, Kangcheng, Shanghai, China).

### Cell counting Kit-8 (CCK-8) assay

Initially, 1 × 10^4^ hBMSCs and HOS cells were plated in a well of the 96-well plate and cultured for 1, 2 or 3 days at 37 °C in a humidified atmosphere of 5% CO_2_. Every 24 h, cells were incubated with 100µL of medium containing 10 µL CCK-8 reagent (#C0037, Beyotime) for 2 h at 37 °C. Next, optical density (OD) values were identified at the excitation wavelength of 450 nm to determine cell proliferation rate. The proliferation rate was normalized against the control cells.

### Transwell assay

Transwell chambers with 8 μm pore polycarbonate membrane inserts (#REF353097, BD Biosciences, SanJose, CA, USA) were used. In transwell assay for invasion but not migration, 50mL Matrigel (#356234, BD Biosciences) was coated on the inner side of the inserts. After starvation by incubation with FBS-free medium for 12 h, hBMSCs and HOS cells were resuspended and added to the upper chambers, while 0.5mL/well cell growth medium was added to the lower chambers. After 24 h, the transwell chambers were fixed with 4% PFA at 25 °C for 30 min, and the Matrigel was wiped off with a cotton swab in transwell assay for invasion. Crystal violet was added to the Transwell chambers to dye cells at 25 °C for 30 min. Finally, the number of crystal violet-dyed cells in the lower chamber was manually counted under a microscope (Eclipse Ci-L, Nikon).

### Nude mice xenograft tumor model

Six-week-old male nude mice were adaptively fed for 3 days. Then 15 mice were randomly divided into 3 groups (5 mice per group). The food intake was given to each subject standardized during this study. Next, mice were treated with subcutaneous injection of 2 × 10^6^ hsa_circ_0003611-silenced hBMSCs and 2 × 10^6^ HOS cells into right dorsal flanks. After subcutaneous injection, tumor volume in mice was measured at the duration of day 3, 6, 9, 12, 15, 18, 21, 24, 27 and 30. Subsequently, the mice were anesthetized with 50 mg/kg of pentobarbital and euthanized using 30% v/min CO_2_ at day 30 after injection in accordance with The Guide for the Care and Use of Laboratory Animals 8th Edition and AVMA Guidelines for Euthanasia (2013), and tumor size and tumor weight were measured. All animal experiments were approved by the Animal Ethics Committee of Zhongshan City People’s Hospital.

### Immunocytochemistry (IHC)

Sections of xenograft tumors derived from nude mice underwent permeabilization by phosphate buffered saline (PBS) containing 0.2% Triton X-100 (PBST) 1 h at 25 °C. Then sections were washes and blocked by PBS containing 1% BSA, 5% goat serum and 0.1% Triton X-100 in PBS for 1 h at 25 °C followed by the incubation with MYC antibody (1:200, #67447-1-Ig, Proteintech), Tbx3 antibody (1:200, #16741-1-AP, Proteintech) or SATB homeobox 2 (SATB2) antibody (1:200, #CSB-PA892170LA01HU, CUSABIO, Wuhan, Hubei, China) overnight at 4 °C. After washing, sections were incubated with biotin-labeled secondary antibodies for 1 h at 25 °C. Subsequently, sections were washed and stained by DAB Substrate Kit (DAB staining) (#AR1027, Boster, Wuhan, Hubei, China) before being photographed using a microscope (Eclipse Ci-L, Nikon).

### RNA Immunoprecipitation (RIP)

Initially, hBMSCs were collected and lysed followed by the interruption of nucleic acid fragments using ultrasound. Then cell lysate was incubated with 1 µg IGF2BP3 antibody (#14642-1-AP, Proteintech) at 4 °C overnight. The next day IGF2BP3 antibody was captured by avidin magnetic beads, and protein-binding RNA fragments were eluted and determined using qRT-PCR. The primers used for qRT-PCR were listed in Table 1.

### RNA antisense purification (RAP)

Firstly, biotin (Bio)-labeled hsa_circ_0003611 probe was synthetized by Hytran Biotech (Guangzhou, Guangdong, China) and the sequences of probes were: hsa_circ_0003611: 5′Bio-GCCAGAGGACUAUGAGAAUG-3′; Bio-NC (negative control): 5′Bio-UUGUACUACACAAAAGUACU-3′. Then hBMSCs were cross-linked using 1% formaldehyde at 25 °C for 10 min and stopped by glycine at 25 °C. Next, hBMSCs were lysed to shear DNA utilizing DNase at 37 °C for 10 min. Subsequently, lysates were incubated with Bio-labeled probes and streptavidin magnetic beads at 25 °C for 30 min followed by the incubation with DNA-free cell lysis buffer at 45 °C for 180 min. Then precipitated complexes were captured by a magnetic separation device (Thermo Fisher Scientific, Waltham, MA, USA). Finally, immunoprecipitated RNAs were detected by qRT-PCR after elution.

### RNA decay assay

First, hBMSCs were treated with 5 µg/mL actinomycin D (#JP-6220, Jinpin Chemical Biotech, Shanghai, China) to hinder DNA transcription. Then hBMSCs were collected at 0, 1, 2, 3, 4, and 5 h post actinomycin D treatment, and MYC mRNA level was detected by qRT-PCR to generate the decline curve through linear regression by taking the time post actinomycin D treatment as the abscissa and the MYC mRNA as the ordinate. Subsequently, the half-life (t_1/2_) of MYC mRNA level was calculated according to the following formula: 0.693\slope of decline curve. When the slope of decline curve is negative, the formula is -0.693\slope decline curve. The primers used for qRT-PCR were present in Table 1.

### Bioinformatic analysis

The m^6^A sites on hsa_circ_0003611 were predicted by RMVar 2.0 (https://rmvar.renlab.cn/#/home).

### Methylated RNA Immunoprecipitation (MeRIP)

Initially, hBMSCs were collected and lysed followed by the interruption of nucleic acid fragments using ultrasound. Then hBMSC lysate was incubated with 1 µg m^6^A antibody (#ab208577, Abcam) at 4 °C overnight. Next, the m^6^A antibody was captured by avidin magnetic beads, while m^6^A-modified hsa_circ_0003611 was eluted and detected by qRT-PCR. The primers used for qRT-PCR were listed in Table 1.

### RNA pulldown

The specific biotinylated probes for wild type (WT) hsa_circ_0003611 or that with mutated m^6^A sites (MUT) and negative control (NC) probes were synthesized by Hytran Biotech (Guangzhou, Guangdong, China). Then MSCs were collected and lysed. After removing deposition by centrifuge, 100µL supernatant was collected for input, and the rest supernatant was incubated with biotinylated probes and streptavidin magnetic beads (#65605D, Thermo Fisher Scientific) for 2 h at 25 °C with gentle shaking. Next, beads were washed and magnetically separated, and then proteins binding with hsa_circ_0003611 were eluted and analyzed by WB using IGF2BP3 antibody (1:1000, #ab177477, Abcam). GAPDH was set as the NC in this experiment.

### Statistical analysis

Quantitative data were present as mean ± standard deviation (SD). SPSS 20 software (Chicago, IL, USA) was utilized for the analysis of statistical differences. Briefly, the comparison between the two groups was analyzed by the unpaired Student’s t-test, while the post-hoc Tukey’s test following One way ANOVA was applied for the statistics among multiple groups. *P* < 0.05 was considered as statistically significant.

## Results

### Hsa_circ_0003611 level is absent in OS cells compared to that in osteoblasts and MSCs

**Fig. 1:**
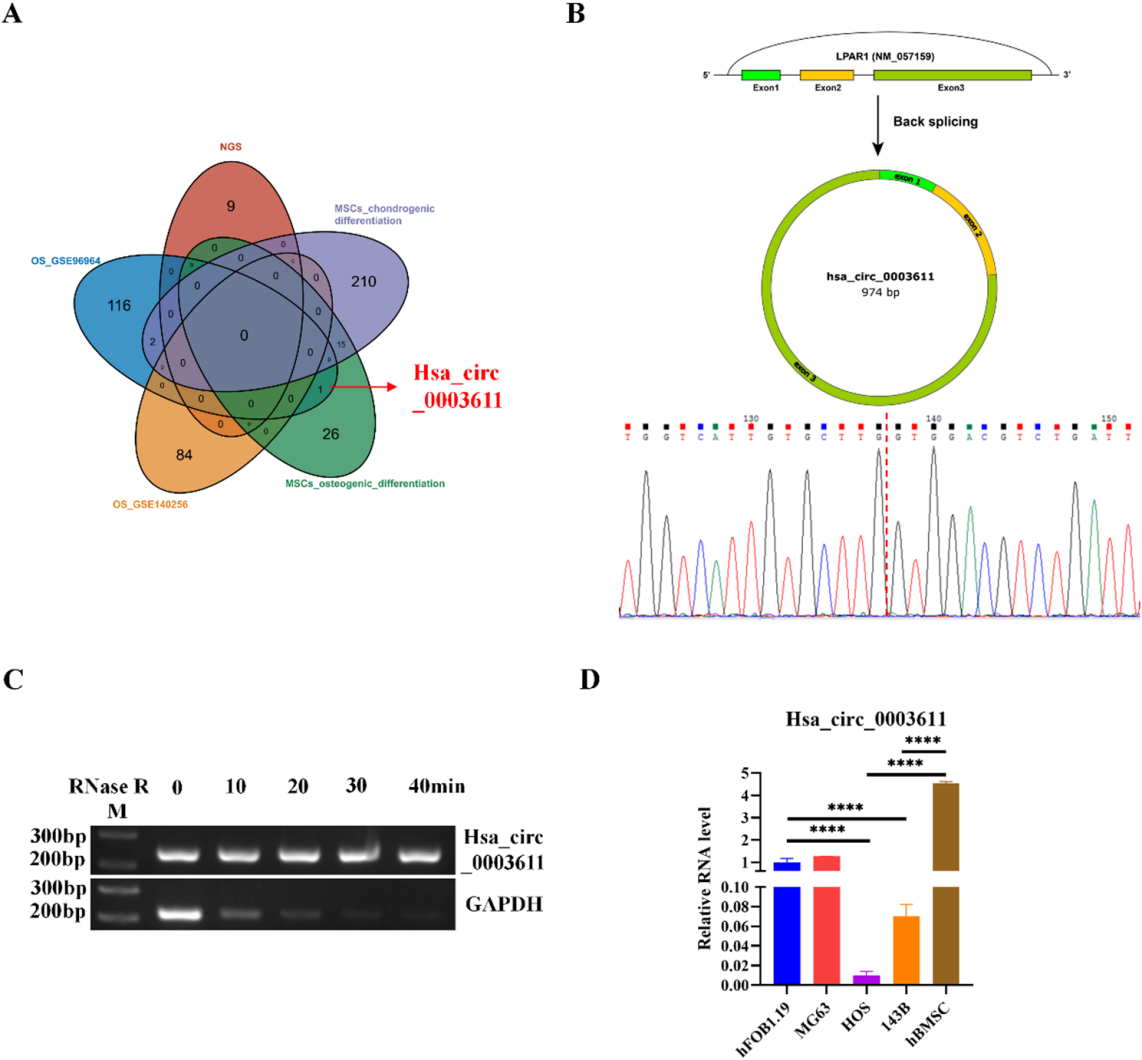
Hsa_circ_0003611 level is absent in OS cells compared to that in osteoblasts and MSCs. **A** The overlap of differentially expressed circRNAs in OS and circRNAs contributing to the differentiation MSCs into osteoblasts. **B** The schematic diagram show generation of hsa_circ_0003611. Further Sanger sequencing confirmed that PCR product spanned the circular junction of predicted hsa_circ_0003611. The magenta dash line indicates the circular junction site. **C** PCR detection of hsa_circ_0003611 level in hBMSCs after RNase R treatment. **D** Hsa_circ_0003611 level in hFOB1.19, 143B, HOS cells and hBMSCs. *N* = 3. **P < 0.01, ****P < 0.0001

Gene expression profiles of circRNAs in OS tissues or cell lines were obtained from GEO DataSets (GSE96964 and GSE140256) and a previous study [22] to screen differentially expressed circRNAs in OS tissues or cell lines. Besides, circRNAs involved in the differentiation MSCs into osteoblasts have been studied [23]. Overlap of differentially expressed circRNAs in OS and circRNAs contributing to the differentiation MSCs into osteoblasts found that hsa_circ_0003611 is both differentially expressed in OS cells and involved in the differentiation MSCs into osteoblasts (Fig. 1A). Further Sanger sequencing confirmed that the PCR product spanned the circular junction for hsa_circ_0003611 in hBMSCs (Fig. 1B). Moreover, hsa_circ_0003611 level in hBMSCs did not change after RNase R treatment, whereas GAPDH mRNA level was significantly declined in hBMSCs treated with RNase R (Fig. 1C). These results suggest that hsa_circ_0003611 forms a circular RNA and is stably expressed. Additionally, results of qRT-PCR showed that hsa_circ_0003611 level was close to zero in 143B, HOS cells compared to that in hFOB1.19 cells and hBMSCs (Fig. 1D). Above data suggest that hsa_circ_0003611 level is absent in OS cells compared to that in osteoblasts and MSCs.

### Silence of hsa_circ_0003611 facilitates the transformation of MSCs into OS cells in vitro

**Fig. 2:**
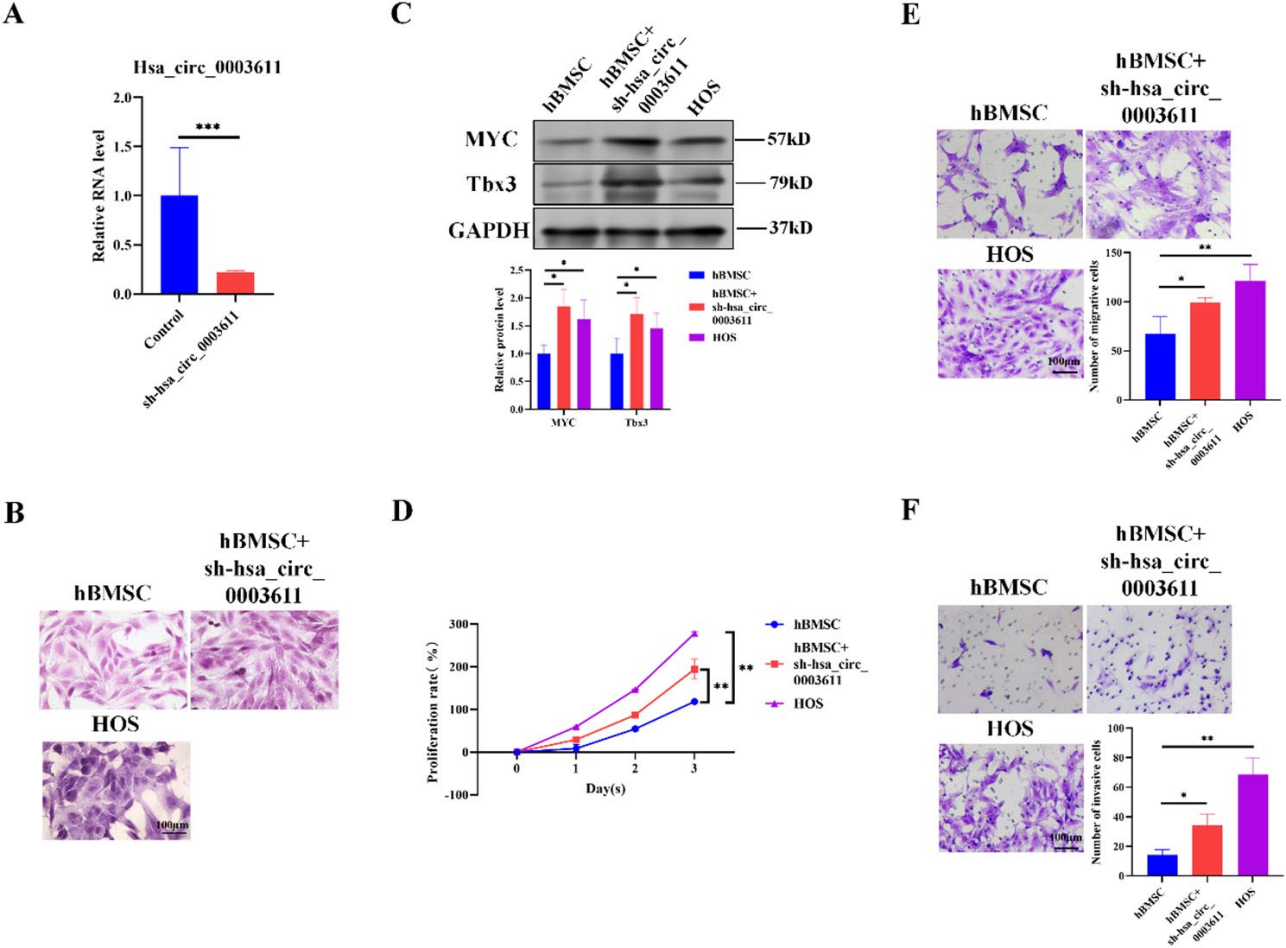
Hsa_circ_0003611 silence enhances the transformation of MSCs into OS cells in vitro. **A** Hsa_circ_0003611 level in hBMSCs infected with or without hsa_circ_0003611 shRNA. **B** Images of H&E staining performed in hBMSCs infected with or without hsa_circ_0003611 shRNA. Bar = 100 μm. **C** Protein levels of MYC and Tbx3 in hBMSCs infected with or without hsa_circ_0003611 shRNA. **D** The proliferation rate of hBMSCs infected with or without hsa_circ_0003611 shRNA. **E** Images of Transwell assay for migration performed in hBMSCs infected with or without hsa_circ_0003611 shRNA. Bar = 100 μm. **F** Images of Transwell assay for invasion performed in hBMSCs infected with or without hsa_circ_0003611 shRNA. Bar = 100 μm. *N* = 3. sh-hsa_circ_0003611: hBMSCs with hsa_circ_0003611 stably silenced. *P < 0.05, **P < 0.01, ****P < 0.0001

To identify the effect of hsa_circ_0003611 on the transformation of MSCs into OS cells, hBMSCs with hsa_circ_0003611 stably silenced (sh-hsa_circ_0003611) was constructed by shRNA (Fig. 2A). H&E staining found that the phenotype of hsa_circ_0003611-silenced hBMSCs was similar to that of OS cell line HOS cells (Fig. 2B). Besides, silence of hsa_circ_0003611 increased levels of markers for OS in hBMSCs, including MYC and Tbx3 (Fig. 2C). Levels of MYC and Tbx3 in hsa_circ_0003611-silenced hBMSCs approached to those in HOS cells (Fig. 2C).

Moreover, results of CCK-8 assay showed that hsa_circ_0003611 silence improved hBMSC proliferation rate closing to that of HOS cells (Fig. 2D). Additionally, transwell assay indicated that silence of hsa_circ_0003611 enhanced the migration and invasion capabilities of hBMSCs closing to those of HOS cells (Fig. 2E, F). Above results suggest that hsa_circ_0003611 silence enhances the transformation of MSCs into OS cells in vitro.

### Hsa_circ_0003611 silence induces the tumorigenicity of MSCs for OS in vivo

**Fig. 3:**
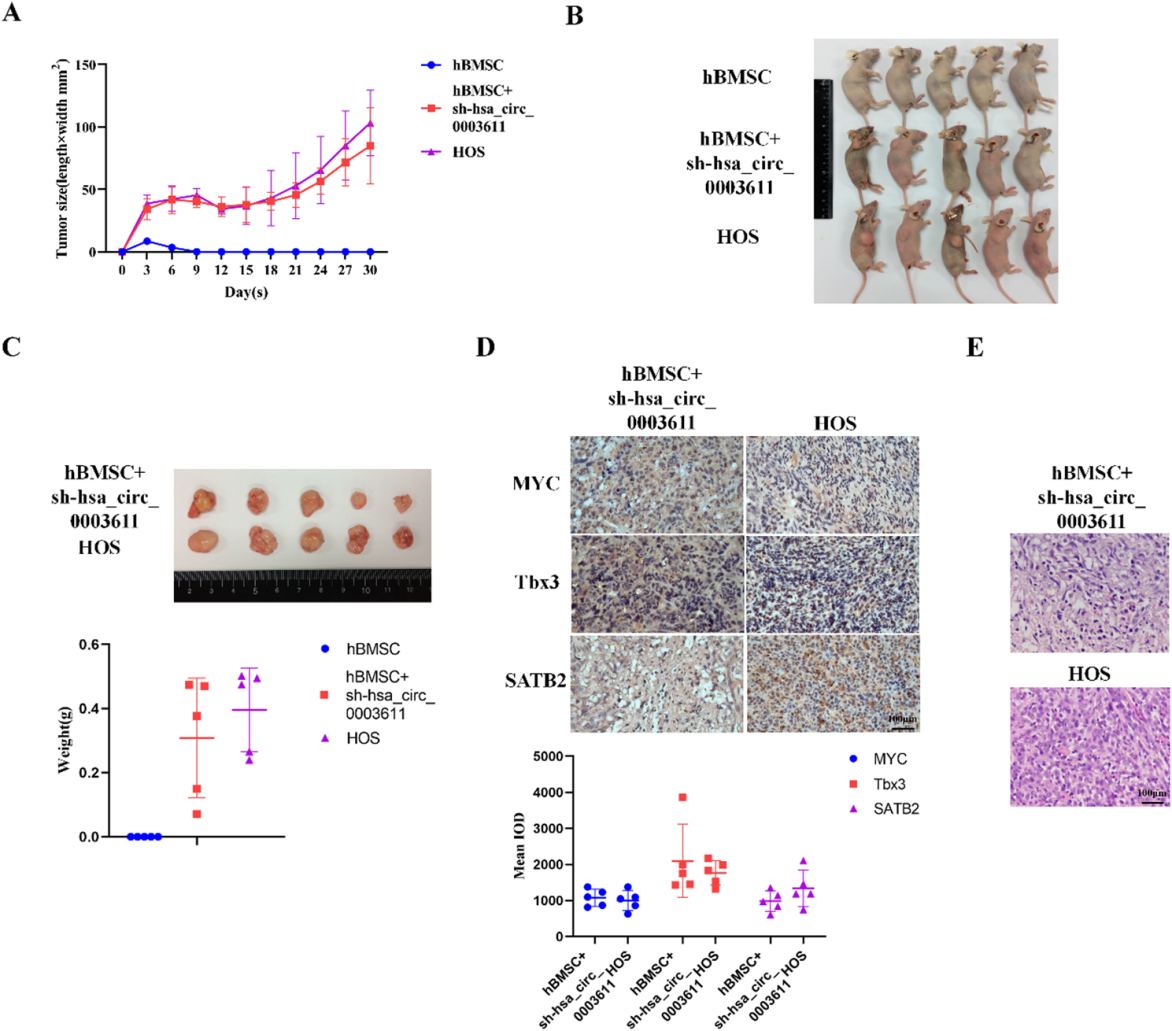
Silence of hsa_circ_0003611 triggers tumorigenicity of MSCs for OS in vivo. **A** Volume of xenograft tumors formed in nude mice injected with hBMSCs, hsa_circ_0003611-silenced hBMSCs and HOS cells. **B** Images of nude mice injected with hBMSCs, hsa_circ_0003611-silenced hBMSCs and HOS cells. **C** Images of xenograft tumors derived from nude mice injected with hBMSCs, hsa_circ_0003611-silenced hBMSCs and HOS cells. The histogram indicates the quantification of tumor weights. **D** Images of IHC performed by MYC, Tbx3 and SATB2 antibodies in xenograft tumors derived from nude mice injected with hsa_circ_0003611-silenced hBMSCs and HOS cells. Bar = 100 μm. **E** Images of H&E staining performed in xenograft tumors derived from nude mice injected with hsa_circ_0003611-silenced hBMSCs and HOS cells. Bar = 100 μm. *N* = 5. sh-hsa_circ_0003611: hBMSCs with hsa_circ_0003611 stably silenced

Subsequently, the tumorigenic potential of hsa_circ_0003611-silenced MSCs in vivo was determined. Results indicated that tumors formation was observed and collected in mice injected with hsa_circ_0003611-silenced hBMSCs, yet the tumor volume and weight were much smaller than those in mice injected with HOS cells (Fig. 3A–C). Moreover, there was no tumor observed and collected in mice injected with control hBMSCs (Fig. 3A–C).

Additionally, tumors developing from hsa_circ_0003611-silenced MSCs exhibited the presence of OS markers including MYC, Tbx3 and SATB2, which were closed to those in tumors developing from HOS cells (Fig. 3D). Furthermore, results of H&E staining demonstrated that no advanced OS tissue was observed in tumors developing from hsa_circ_0003611-silenced hBMSCs while typical advanced OS tissues were found in tumors developing from HOS cells (Fig. 3E). These results suggest that silence of hsa_circ_0003611 triggers tumorigenicity of MSCs for OS in vivo. It is noteworthy that silence of hsa_circ_0003611 induces the tumorigenicity of MSCs for OS at an early stage.

### Hsa_circ_0003611 suppresses MYC expression by IGF2BP3 in MSCs

**Fig. 4:**
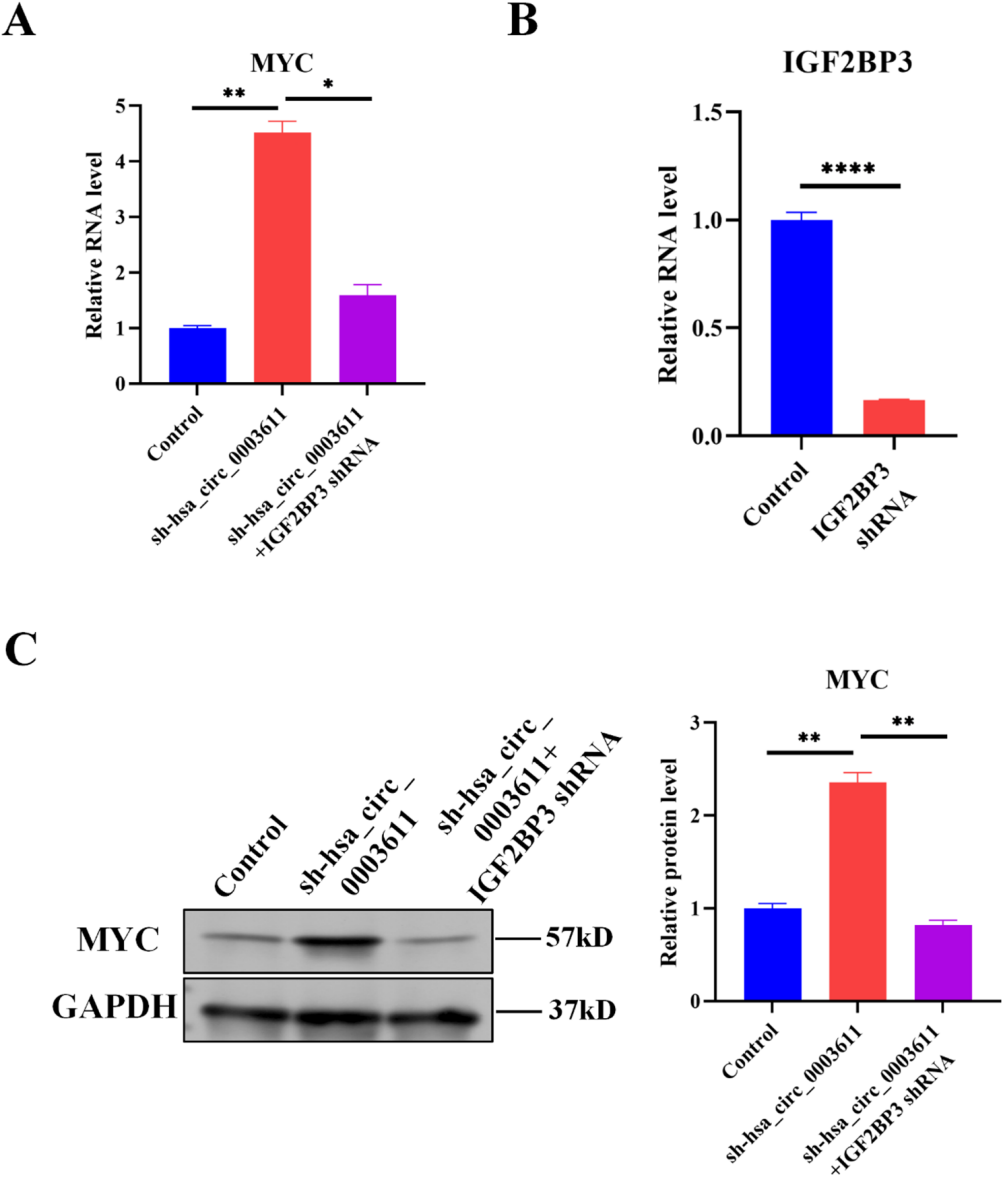
Hsa_circ_0003611 inhibits MYC expression by IGF2BP3 in MSCs. **A** MYC mRNA level in control hBMSCs and hBMSCs with hsa_circ_0003611 stably silenced infected with or without IGF2BP3 shRNA. **B** IGF2BP3 mRNA level in hBMSCs infected with or without IGF2BP3 shRNA. **C** MYC protein level in control hBMSCs and hBMSCs with hsa_circ_0003611 stably silenced infected with or without IGF2BP3 shRNA. sh-hsa_circ_0003611: hBMSCs with hsa_circ_0003611 stably silenced. *P < 0.05, **P < 0.01, ****P < 0.0001

As overexpression of MYC would induce the transformation of MSCs into OS cells [11, 12], and our results indicate that silence of hsa_circ_0003611 elevates MYC protein level in hBMSCs (Fig. 2C). Then the effect of hsa_circ_0003611 on MYC mRNA was also determined. Results of qRT-PCR revealed that silence of hsa_circ_0003611 also increased MYC mRNA level in hBMSCs (Fig. 4A). MYC-induced nuclear antigen 53 (Mina53) and dyskeratosis congenita 1 (DKC1) are known MYC target genes [24, 25]. Hsa_circ_0003611 silence elevated Mina53 (Supplementary Figure S1A) and DKC1 (Supplementary Figure S1B) mRNA levels in hBMSCs as well. Above results suggest that hsa_circ_0003611 represses MYC expression in MSCs.

Additionally, it has been reported that IGF2BP3 enhances MYC mRNA stability [18]. Results of qRT-PCR also demonstrated that silence of IGF2BP3 by shRNA (Fig. 4B) reversed the effect of hsa_circ_0003611 silence on MYC mRNA level (Fig. 4A) and protein level (Fig. 4C) in hBMSCs. Thus, these data suggest that hsa_circ_0003611 inhibits MYC expression by IGF2BP3 in MSCs.

### Silence of hsa_circ_0003611 enhances the transformation of MSCs into OS cells by activating MYC

**Fig. 5:**
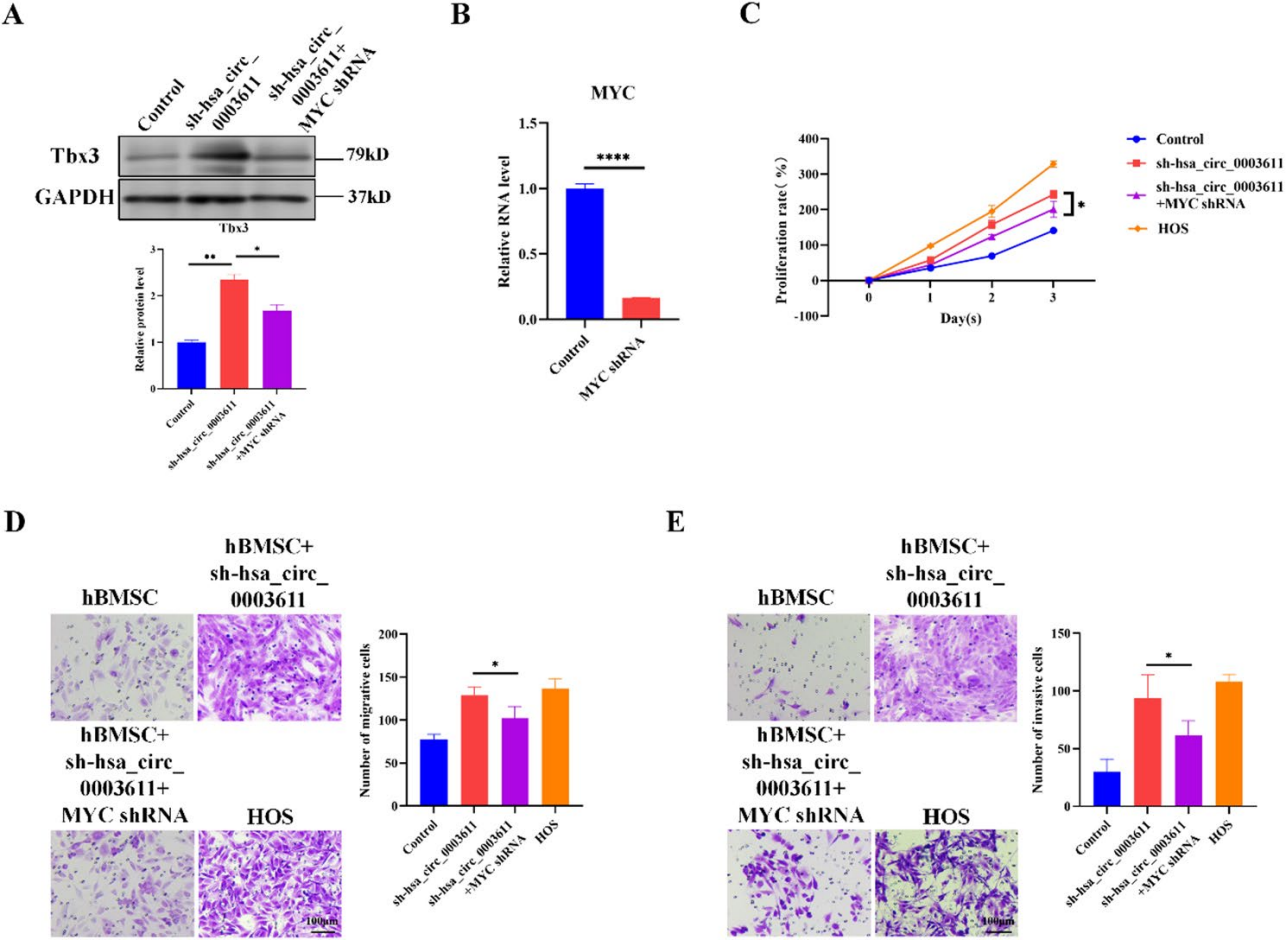
Silence of hsa_circ_0003611 promotes the transformation of MSCs into OS cells by activating MYC. **A** Tbx3 protein level in control hBMSCs and hBMSCs with hsa_circ_0003611 stably silenced infected with or without MYC shRNA. **B** The MYC mRNA level in hBMSCs infected with or without MYC shRNA. **C** The proliferation rate of control hBMSCs and hBMSCs with hsa_circ_0003611 stably silenced infected with or without MYC shRNA. **D** Images of Transwell assay for migration performed in control hBMSCs and hBMSCs with hsa_circ_0003611 stably silenced infected with or without MYC shRNA. Bar = 100 μm. **E** Images of Transwell assay for invasion performed in control hBMSCs and hBMSCs with hsa_circ_0003611 stably silenced infected with or without MYC shRNA. Bar = 100 μm. *N* = 3. sh-hsa_circ_0003611: hBMSCs with hsa_circ_0003611 stably silenced. *P < 0.05, ****P < 0.0001

Next, whether hsa_circ_0003611 facilitated the transformation of MSCs into OS cells by MYC was identified. Except MYC (Fig. 4C), silence of hsa_circ_0003611 also increased Tbx3 protein level in hBMSCs (Fig. 5A), whereas silence of MYC by shRNA (Fig. 5B) abolished the effect of hsa_circ_0003611 silence on Tbx3 protein level (Fig. 5A). Besides, CCK-8 assay indicated that hsa_circ_0003611 silence improved hBMSC proliferation rate closing to that of HOS cells yet silence of MYC reversed the effect of hsa_circ_0003611 silence on hBMSC proliferation rate (Fig. 5C). Additionally, transwell assay showed that silence of hsa_circ_0003611 enhanced the migration and invasion capabilities of hBMSCs but silence of MYC neutralized the effect of hsa_circ_0003611 silence on hBMSC migration and invasion (Fig. 5D, E). These results together suggest that silence of hsa_circ_0003611 promotes the transformation of MSCs into OS cells by activating MYC.

### Hsa_circ_0003611 decreases MYC mRNA level by disrupting the association between IGF2BP3 and MYC mRNA to decline its stability in MSCs

**Fig. 6:**
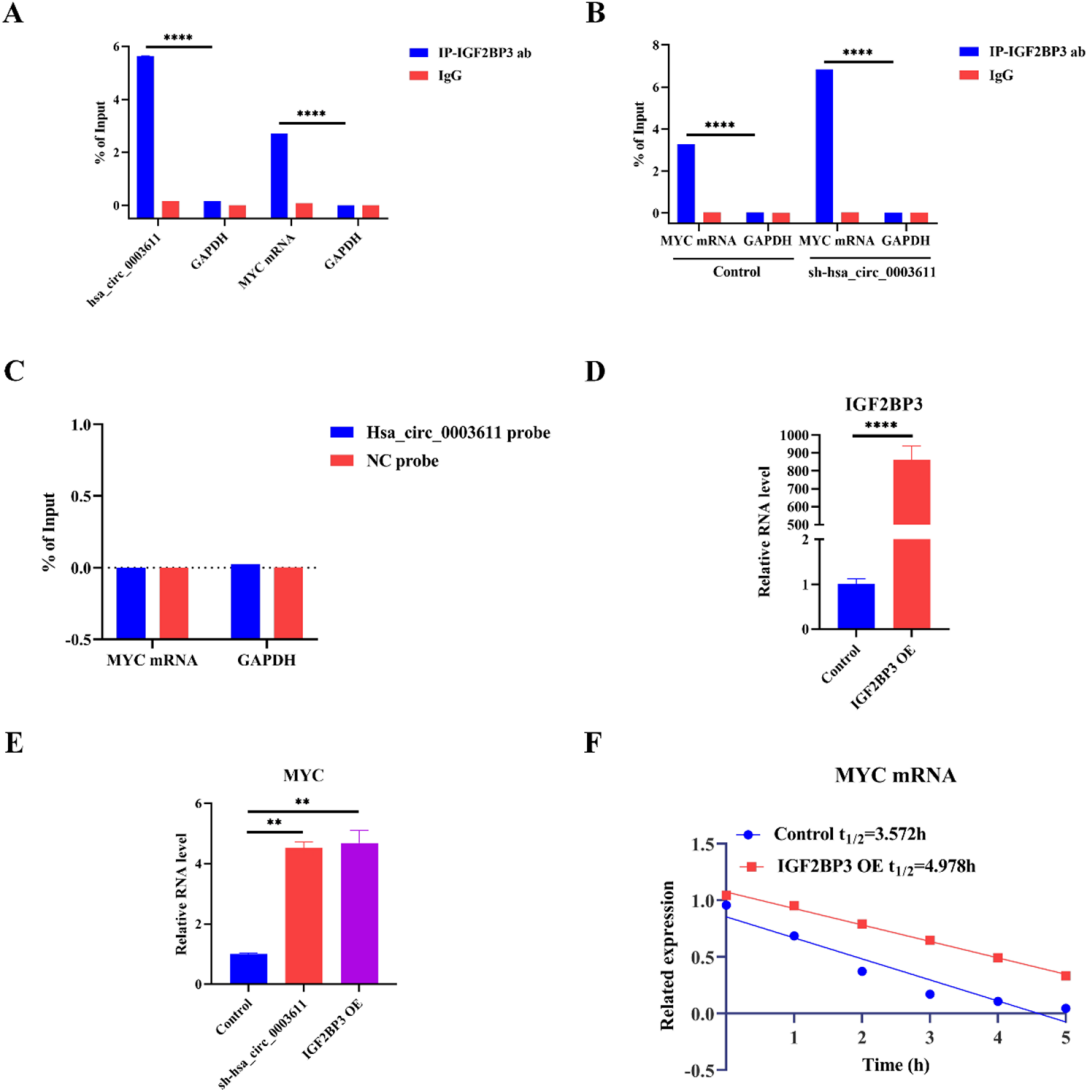
Hsa_circ_0003611 reduces MYC mRNA level by blocking the association between IGF2BP3 and MYC mRNA to decline its stability in MSCs. **A** The level of hsa_circ_0003611 or MYC mRNA bound with IGF2BP3 in hBMSCs detected by RIP. **B** The MYC mRNA level bound with IGF2BP3 in control and sh-hsa_circ_0003611 hBMSCs detected by RIP. **C** Quantification of MYC mRNA by qRT-PCR following RAP performed with Bio-hsa_circ_0003611 probe in hBMSCs. **D** IGF2BP3 mRNA level in hBMSCs transfected with or without IGF2BP3 expression vector. **E** MYC mRNA level in control, sh-hsa_circ_0003611 and IGF2BP3-overexpressed hBMSCs. **F** The MYC mRNA level in control or IGF2BP3-overexpressed hBMSCs at 0 h, 1 h, 2 h, 3 h, 4 h and 5 h post actinomycin D treatment determined by qRT-PCR, and the half-life (t_1/2_) of MYC mRNA in control or IGF2BP3-overexpressed hBMSCs. sh-hsa_circ_0003611: hBMSCs with hsa_circ_0003611 stably silenced; OE: overexpression; NC, negative control. *N* = 3. **P < 0.01, ****P < 0.0001

Then the mechanism of hsa_circ_0003611 suppressing MYC expression by IGF2BP3 was investigated. Results of RIP performed by IGF2BP3 antibody revealed that IGF2BP3 bound to both hsa_circ_0003611 and MYC mRNA in hBMSCs (Fig. 6A). Besides, RIP performed by IGF2BP3 antibody also indicated that silence of hsa_circ_0003611 enhanced the association between IGF2BP3 and MYC mRNA in hBMSCs (Fig. 6B). Moreover, RAP assessed by hsa_circ_0003611 probe indicated that hsa_circ_0003611 could not bind with MYC mRNA in hBMSCs (Fig. 6C). These results suggest that hsa_circ_0003611 disrupts the interaction of IGF2BP3 and MYC mRNA by binding with IGF2BP3 in MSCs.

Additionally, qRT-PCR analysis showed that both hsa_circ_0003611 silence and IGF2BP3 overexpression (Fig. 6D) increased MYC mRNA in hBMSCs (Fig. 6E). As IGF2BP3 could regulate target mRNA level by modulating its stability, the effects of IGF2BP3 on MYC mRNA stability in hBMSCs was identified using RNA decay assay. Results demonstrated that IGF2BP3 overexpression significantly improved the half-life (t_1/2_) of MYC mRNA (4.978 h) compared to that in the control hBMSCs (3.572 h) (Figure. 6 F). Above data suggest that hsa_circ_0003611 reduces MYC mRNA level by blocking the association between IGF2BP3 and MYC mRNA to decline its stability in MSCs.

### m^6^ A modification hinders the association between hsa_circ_0003611 and IGF2BP3 to reduces the effect of hsa_circ_0003611 on the interaction of IGF2BP3 and MYC mRNA in MSCs

**Fig. 7:**
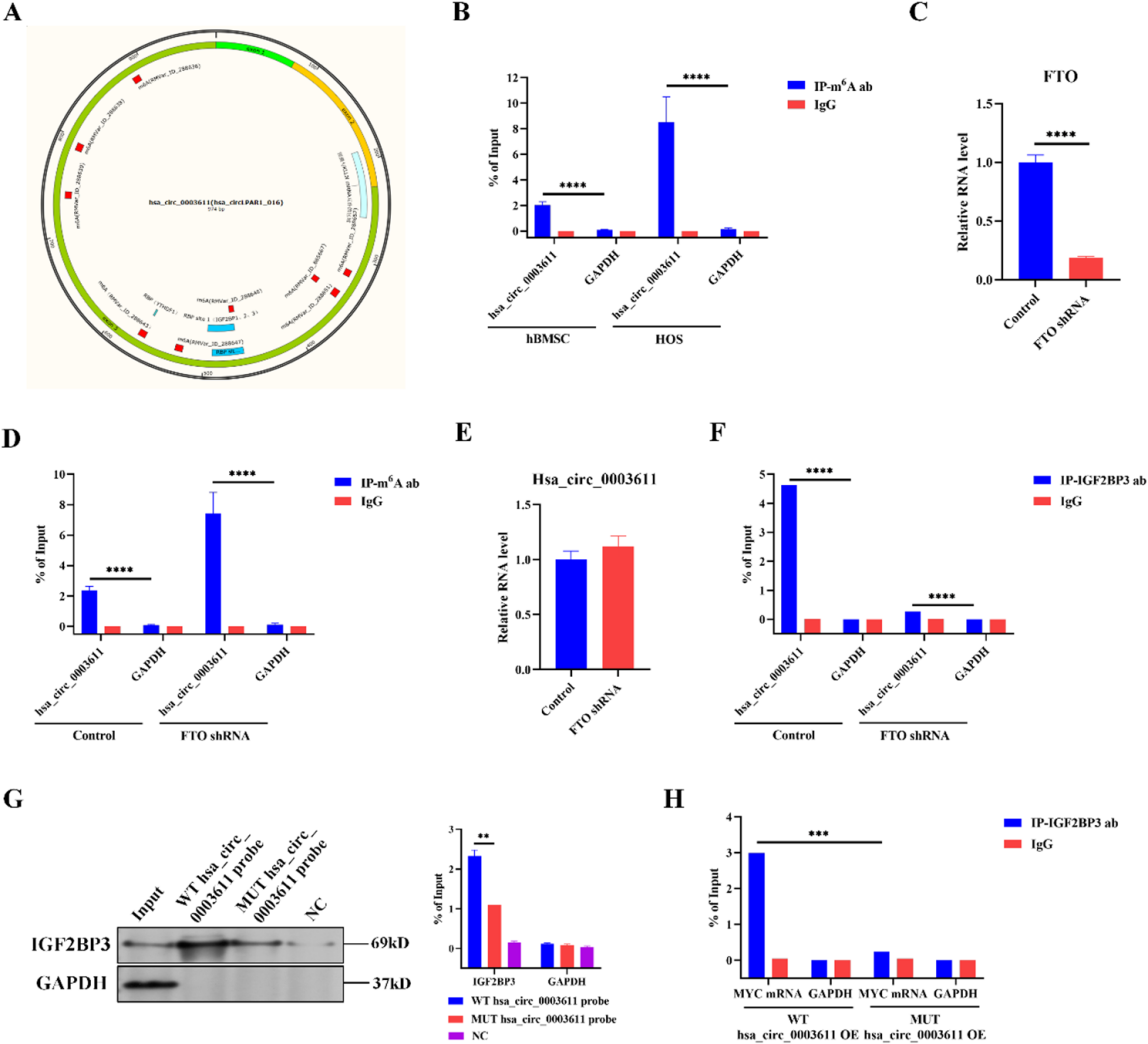
m^6^A modification disrupts the association between hsa_circ_0003611 and IGF2BP3 to decrease the effect of hsa_circ_0003611 on the association between IGF2BP3 and MYC mRNA in MSCs. **A** Bioinformatic analysis for m^6^A sites on hsa_circ_0003611. **B** The m^6^A-modified hsa_circ_0003611 level in hBMSCs and HOS cells. **C** The FTO mRNA level in hBMSCs infected with or without FTO shRNA. **D** The m^6^A-modified hsa_circ_0003611 level in hBMSCs infected with or without FTO shRNA. **E** The hsa_circ_0003611 level in hBMSCs infected with or without FTO shRNA. **F** The level of hsa_circ_0003611 bound with IGF2BP3 in control and FTO-silenced hBMSCs detected by RIP. **G** The IGF2BP3 protein level bound with WT or MUT hsa_circ_0003611 probes detected by RNA pulldown. **H** The level of MYC mRNA bound with IGF2BP3 in WT or MUT hsa_circ_0003611-overexpressed hBMSCs detected by RIP. WT hsa_circ_0003611: wild type hsa_circ_0003611; MUT hsa_circ_0003611: hsa_circ_0003611 with mutated m6A sites; NC: negative control; OE: overexpression. *N* = 3. **P < 0.01, ***P < 0.001, ****P < 0.0001

Bioinformatic analysis found several m^6^A sites on hsa_circ_0003611 (Fig. 7A), suggesting that m^6^A modification might affect the functions of hsa_circ_0003611. Results of MeRIP revealed that the m^6^A-modified hsa_circ_0003611 level was increased in HOS cells compared to that in hBMSCs (Fig. 7B). To determine the effect of m^6^A modification on the functions of hsa_circ_0003611 in MSCs, the m^6^A demethylase FTO was silenced by shRNA in hBMSCs (Fig. 7C). Results of MeRIP confirmed that FTO silence increased m^6^A-modified hsa_circ_0003611 level in hBMSCs (Fig. 7D). However, qRT-PCR analysis showed that FTO silence had no effect on hsa_circ_0003611 level in hBMSCs (Fig. 7E).

Then the role of m^6^A modification on the association between hsa_circ_0003611 and IGF2BP3 in MSCs was investigated. Results of RIP performed by IGF2BP3 uncovered that silence of FTO disrupted the interaction of hsa_circ_0003611 and IGF2BP3 in hBMSCs (Fig. 7F). RNA pulldown performed by hsa_circ_0003611 probes with mutated m^6^A sites revealed that mutation of m^6^A sites on hsa_circ_0003611 blocked the association between hsa_circ_0003611 and IGF2BP3 in hBMSCs (Fig. 7G). To further confirmed the effect of m^6^A modification for hsa_circ_0003611 on the interaction of IGF2BP3 and MYC mRNA in MSCs, the hsa_circ_0003611 expression vector with mutated m^6^A sites was constructed. Then results of RIP performed by IGF2BP3 demonstrated that transfection of WT hsa_circ_0003611 expression vector abolished the interaction of IGF2BP3 and MYC mRNA in hBMSCs, yet transfection of hsa_circ_0003611 expression vector with mutated m^6^A sites had no significantly effect on the binding of IGF2BP3 and MYC mRNA in hBMSCs (Fig. 7H). These results together suggest that m^6^A modification disrupts the association between hsa_circ_0003611 and IGF2BP3 to decrease the effect of hsa_circ_0003611 on the association between IGF2BP3 and MYC mRNA in MSCs.

## Discussion

**Fig. 8:**
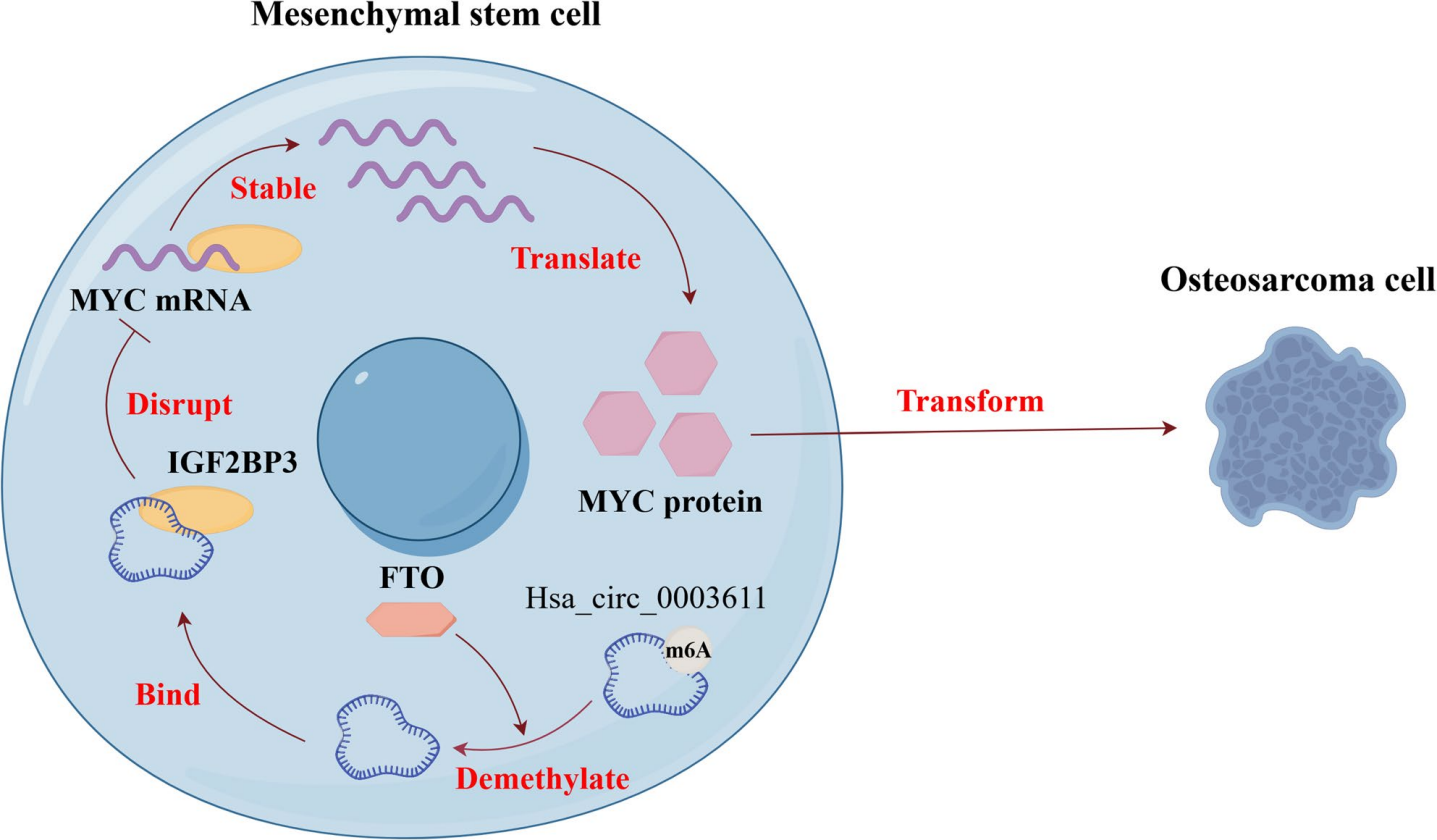
Schematic diagram of molecular mechanisms for the current study. Hsa_circ_0003611 silence enhances the transformation of MSCs into OS cells in vitro and triggers tumorigenicity of MSCs for OS in vivo. Mechanistically, hsa_circ_0003611 inhibits MYC expression by IGF2BP3 in MSCs. Besides, silence of hsa_circ_0003611 promotes the transformation of MSCs into OS cells by activating MYC. Moreover, hsa_circ_0003611 reduces MYC mRNA level by blocking the association between IGF2BP3 and MYC mRNA to decline its stability. Furthermore, m^6^A modification disrupts the association between hsa_circ_0003611 and IGF2BP3 to decrease the effect of hsa_circ_0003611 on the association between IGF2BP3 and MYC mRNA

Our findings suggest that hsa_circ_0003611 level is almost absent in OS cells compared to that in osteoblasts and MSCs. Additionally, hsa_circ_0003611 silence enhances the transformation of MSCs into OS cells in vitro and triggers tumorigenicity of MSCs for OS in vivo. Mechanistically, hsa_circ_0003611 inhibits MYC expression by IGF2BP3 in MSCs. Besides, silence of hsa_circ_0003611 promotes the transformation of MSCs into OS cells by activating MYC. Moreover, hsa_circ_0003611 reduces MYC mRNA level by blocking the association between IGF2BP3 and MYC mRNA to decline its stability. Furthermore, m^6^A modification disrupts the association between hsa_circ_0003611 and IGF2BP3 to decrease the effect of hsa_circ_0003611 on the association between IGF2BP3 and MYC mRNA (Fig. 8).

MYC play a critical role in malignant transformation. For instance, MYC drives the malignant transformation of mature murine B cells into lymphomas by suppressing tumor protein p53 (TP53) activity but activating eukaryotic translation initiation factor 5 A (EIF5A) [26, 27]. Besides, MYC induces the malignant transformation of human bronchial epithelial cells by increasing *colon cancer associated transcript 1* (*CCAT1*) transcription [28]. Our findings and previous studies indicate that MYC induces the transformation of MSCs into OS cells [11, 12]. However, the precise mechanism has not been investigated. Above studies suggest that MYC might drive the transformation of MSCs into OS cells by modulating TP53, EIF5A and CCAT1 activities.

Hsa_circ_0003611 is derived from a non-coding transcript of *lysophosphatidic acid receptor 1* (*LPAR1*). By contrast to hsa_circ_0003611, LPAR1 is upregulated in OS and facilitates OS metastasis [29]. These studies suggest that LPAR1 might be upregulated to enhance the transformation of MSCs into OS cells, and increased production of *LPAR1* coding transcript would lead to reduced generation of *LPAR1* non-coding transcript, resulting in the loss of hsa_circ_0003611 expression during MSC to OS conversion.

A recent study has revealed that m^6^A modification decreases circ-0000953 expression in diabetic nephropathy [30]. Our findings demonstrate that m^6^A-modified hsa_circ_0003611 level is elevated in HOS cells compared to that in hBMSCs. Thus, increased m^6^A modification might be another factor causing the absent of hsa_circ_0003611 expression during the transformation of MSCs into OS cells.

To date, the role of circRNAs in the transformation of MSCs into OS cells is unknown. Our findings suggest that hsa_circ_0003611 silence enhances the transformation of MSCs into OS cells in vitro and triggers tumorigenicity of MSCs for OS in vivo. Thus, this study reports the effect of circRNAs in the transformation of MSCs into OS cells to the best of our knowledge.

The current study indicates that hsa_circ_0003611 decreases MYC mRNA level by blocking the association between IGF2BP3 and MYC mRNA to reduce its stability. Some studies have revealed that circRNAs modulates MYC mRNA stability by RNA binding proteins. Similar to hsa_circ_0003611, circ-hnRNPU binds with non-POU domain containing octamer binding (NONO) protein to decrease MYC mRNA stability in gastric cancer cells [15]. By contrast, hsa_circ_0068631 improves MYC mRNA stability by recruiting eukaryotic translation initiation factor 4A3 (EIF4A3) in breast cancer cells [31]. However, the effect of IGF2BP3 on MYC mRNA stability modulated by circRNAs has not been reported. Thus, the present study demonstrates the regulatory effect of IGF2BP3 on MYC mRNA stability regulated by circRNAs for the first time.

A recent study has found that m^6^A-modified circARHGAP12 improves MYC mRNA stability by directly binding with MYC mRNA via m^6^A-dependent manner in OS [32]. However, our data reveals that m^6^A modification disrupts the association between hsa_circ_0003611 and IGF2BP3 to enhance the interaction of IGF2BP3 and MYC mRNA and increase MYC mRNA stability. Our findings uncover a novel manner of m^6^A-modified circRNA modulating MYC mRNA stability.

IGF2BP3 is a known m^6^A reader [33]. It has been revealed that IGF2BP3 could recognize and bind with m^6^A-modified circRNAs. For example, IGF2BP3 binds with m^6^A-modified circCCAR1 to improve its stability in hepatocellular carcinoma [34]. Similarly, IGF2BP3 recognizes and stabilizes m^6^A-modified circPSMA7 in bladder cancer [35]. By contrast, m^6^A modification disrupts the association between hsa_circ_0003611 and IGF2BP3 in MSCs. Therefore, our data expands the knowledge of IGF2BP3 on regulating m^6^A-modified circRNAs.

However, some limitations were noted. First, the effect of IGF2BP3/MYC axis on the transformation of MSCs into OS cells should be confirmed by in-vivo experiments. Second, the detailed mechanisms underlying MYC-driven transformation of MSCs into OS cells were not investigated. Third, this study could be strengthened by exploring the relationship between hsa_circ_0003611 and the LPAR1 coding transcript.

## Conclusion

In summary, the current study reveals that hsa_circ_0003611 level is almost absent in OS cells compared to that in osteoblasts and MSCs. Besides, our findings suggest that hsa_circ_0003611 hinders the transformation of MSCs into OS cells through inhibiting MYC expression by blocking the association between IGF2BP3 and MYC mRNA to decline MYC mRNA stability. Additionally, m^6^A modification disrupts the interaction of hsa_circ_0003611 and IGF2BP3 to reduce the effect of hsa_circ_0003611 on the association between IGF2BP3 and MYC mRNA. These findings should provide novel targets and strategies for OS treatment.

## Supplementary Information

Below is the link to the electronic supplementary material.

Supplementary Material 1

Supplementary Material 2

## Data Availability

All data are available upon reasonable request to the corresponding author.
