# Supplementary figures and images for: Hsa_circ_0003611 hinders the transformation of mesenchymal stem cells into osteosarcoma cells through suppressing MYC by IGF2BP3 via m6A modification
Source: Biol Res. 2025 Nov 29;59:2. doi: 10.1186/s40659-025-00659-6 (PMC12771901; doi:10.1186/s40659-025-00659-6)

**Figure 2C**

**MYC**

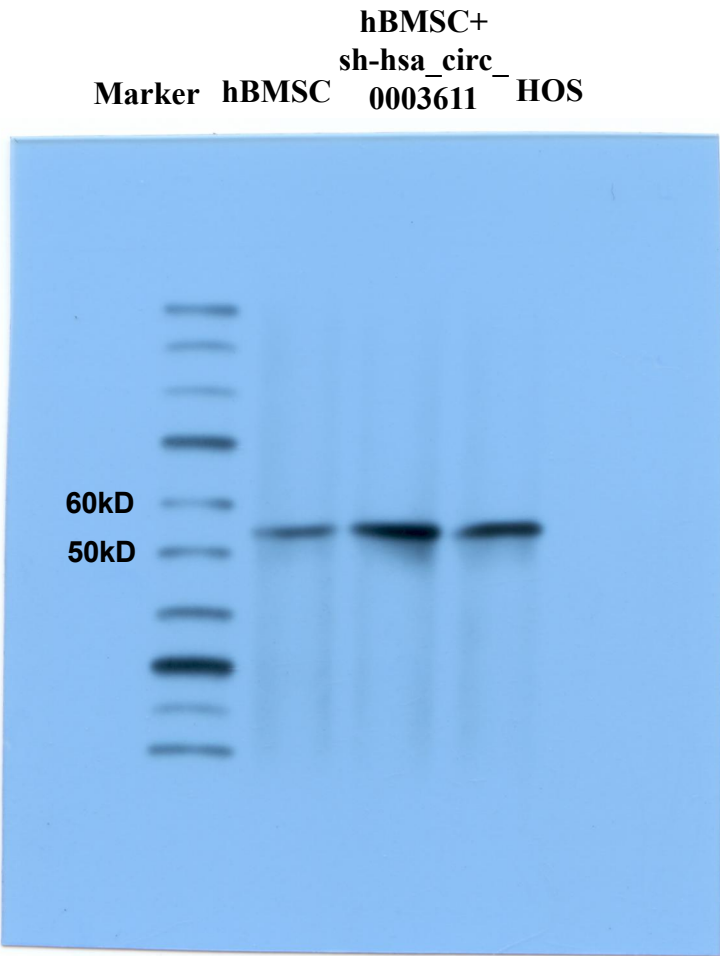

**Tbx3**

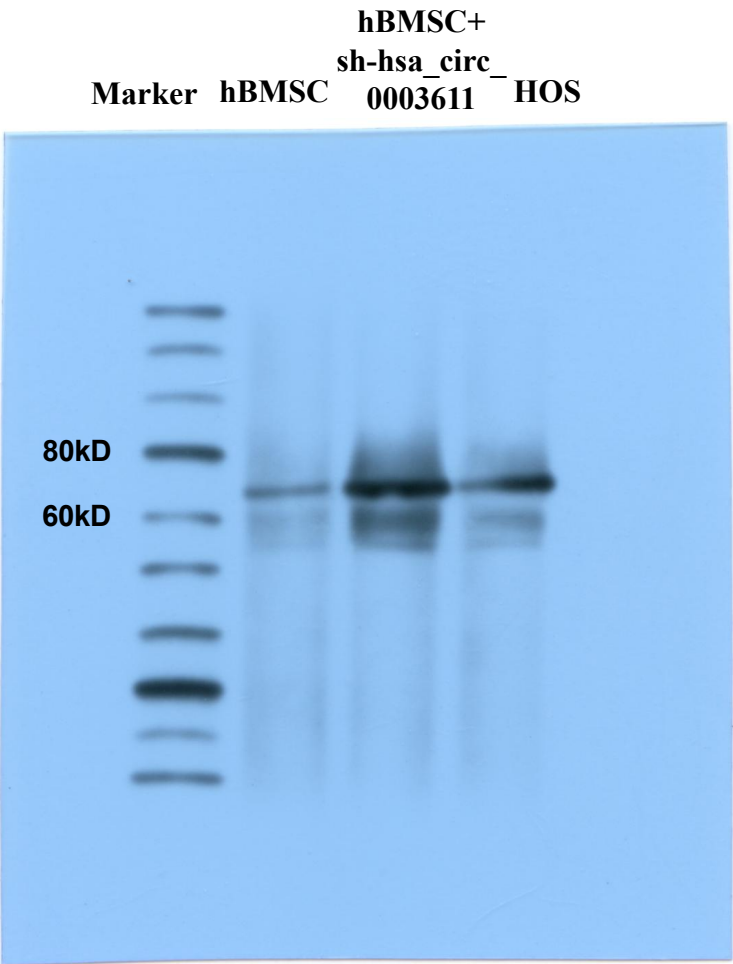

**GAPDH**

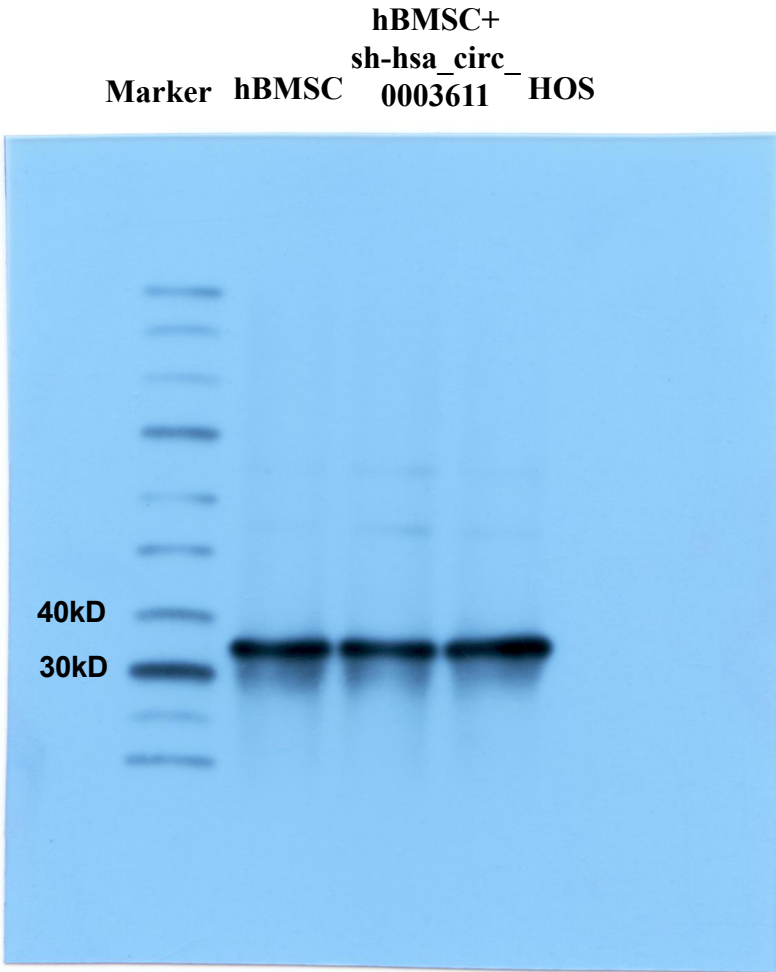

Figure 4C

MYC

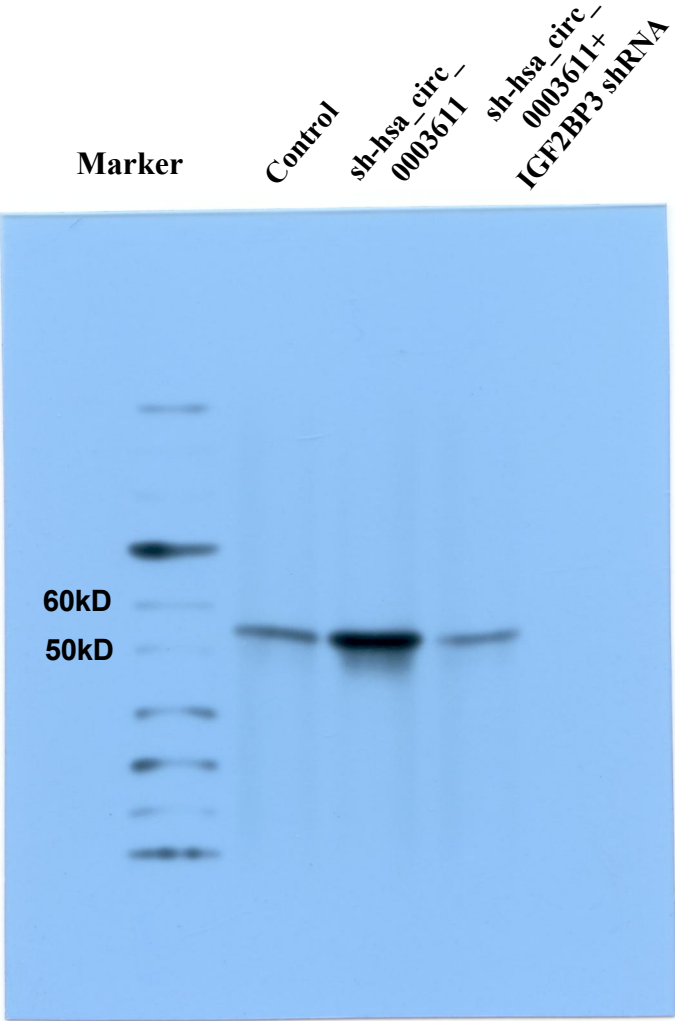

GAPDH

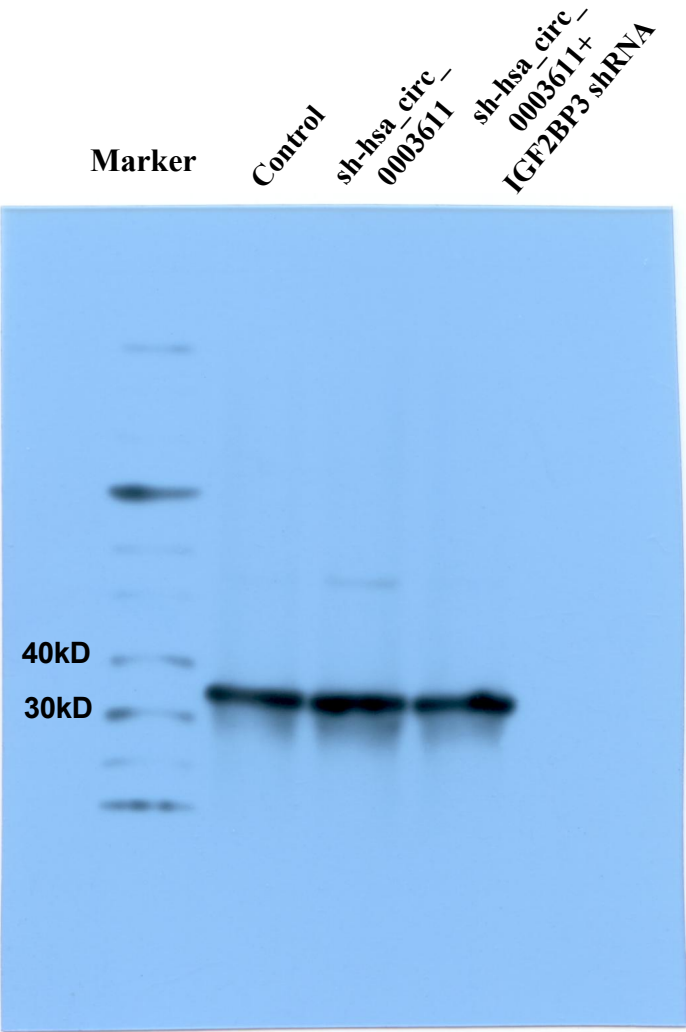

Figure 5A

Tbx3

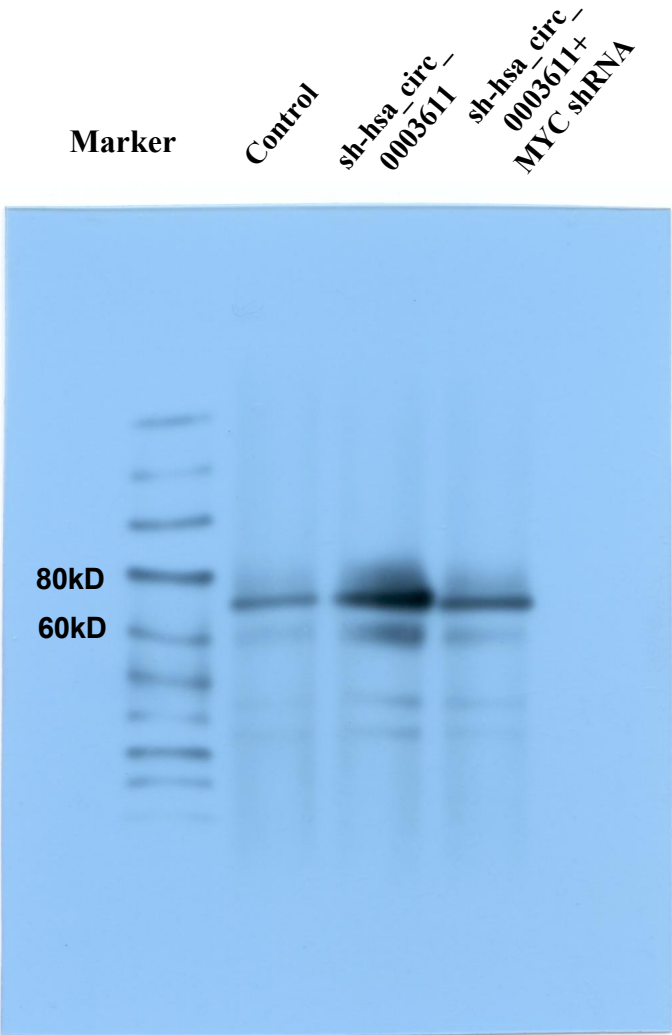

GAPDH

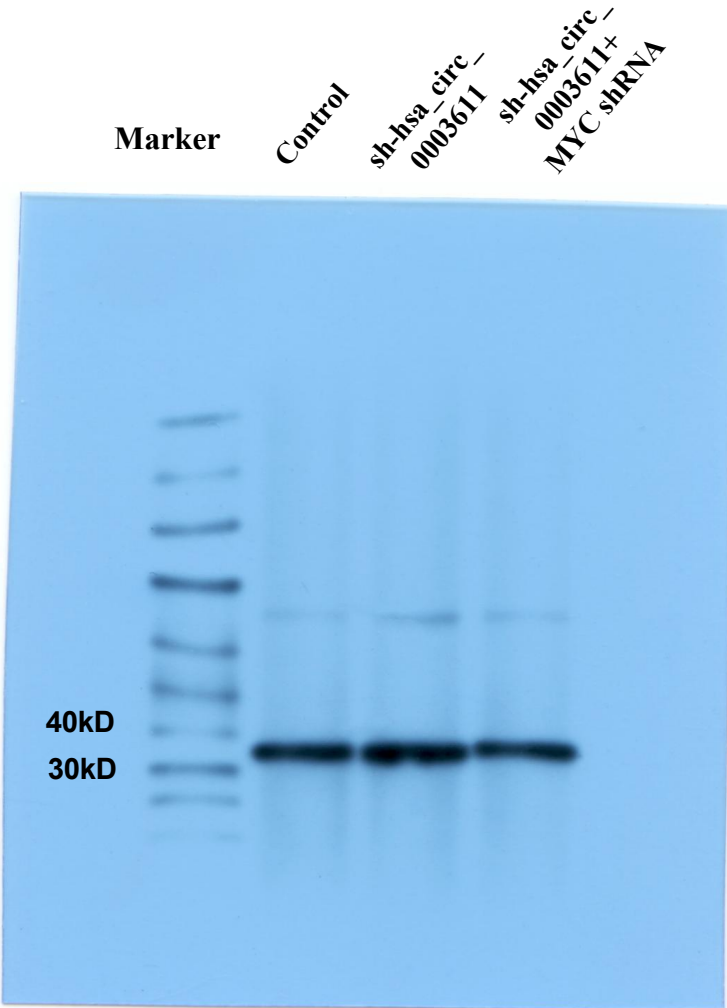

Figure 7G

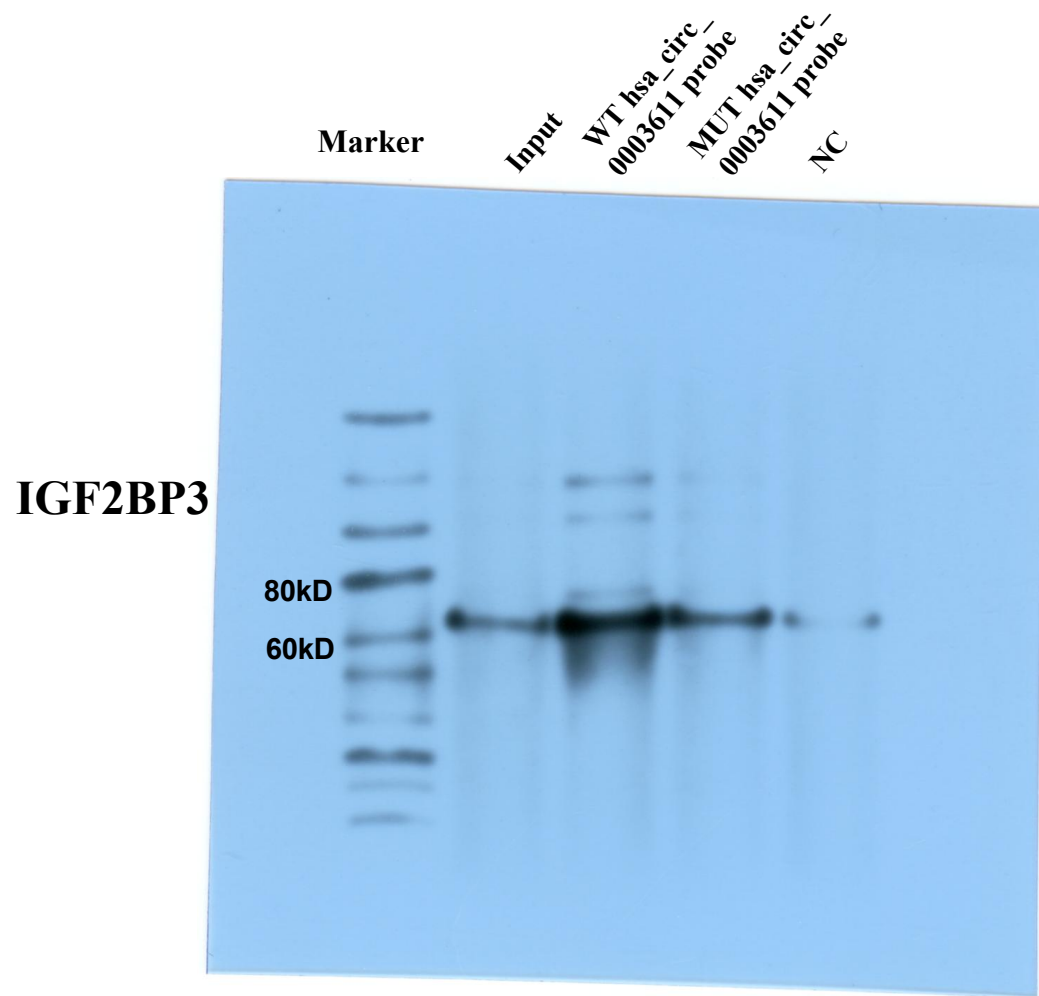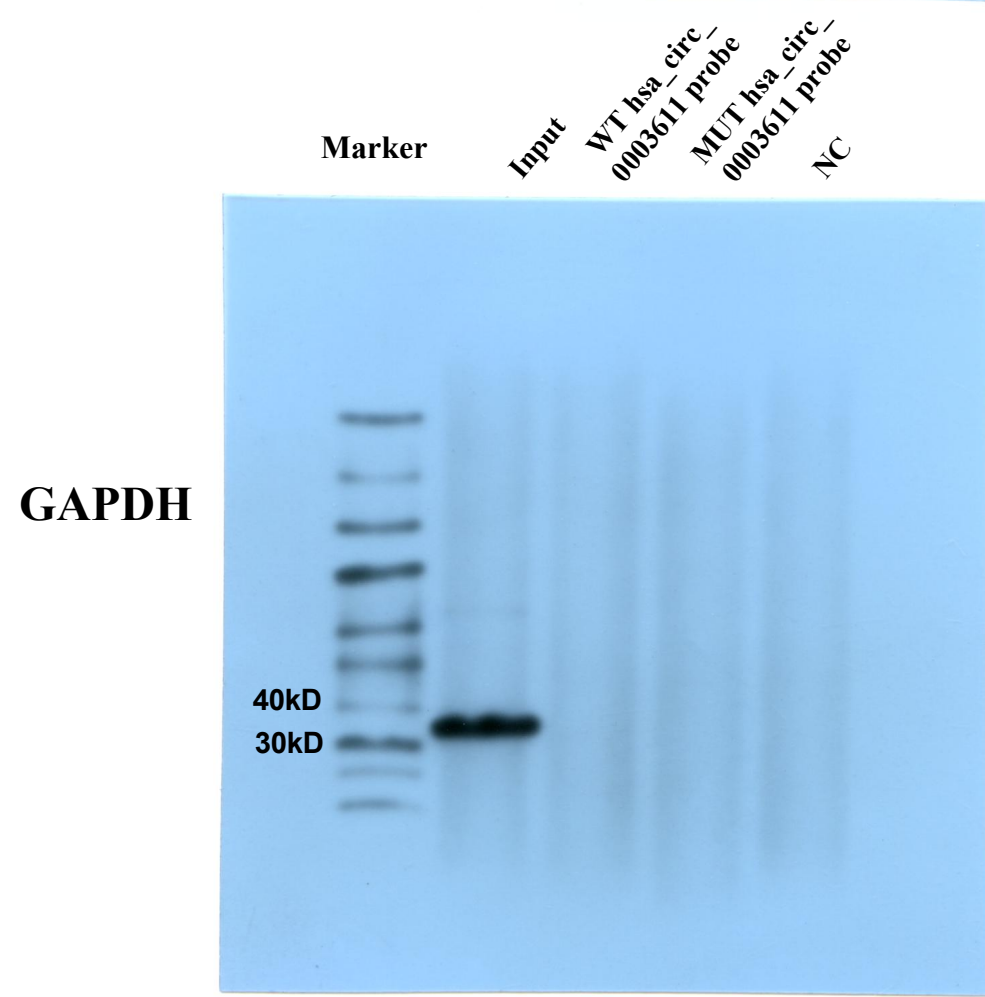

Supplement: Supplementary file 1 — Supplementary Material 1 [file 40659_2025_659_MOESM1_ESM.pdf]
