# Supplementary material for: Hsa_circ_0003611 hinders the transformation of mesenchymal stem cells into osteosarcoma cells through suppressing MYC by IGF2BP3 via m6A modification
Source: Biol Res. 2025 Nov 29;59:2. doi: 10.1186/s40659-025-00659-6 (PMC12771901; doi:10.1186/s40659-025-00659-6)
Supplement: Supplementary file 2 — Supplementary Material 2 [file 40659_2025_659_MOESM2_ESM.docx]

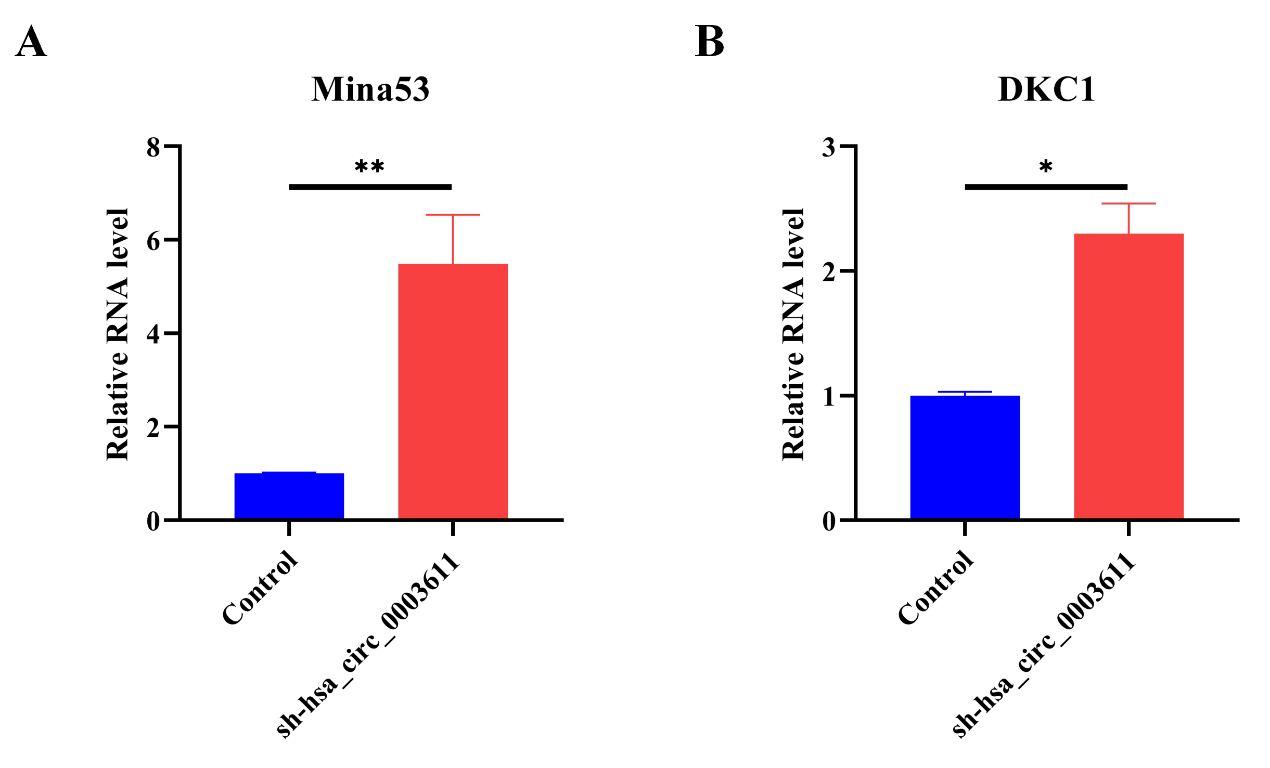


**Supplementary Figure S1 Hsa_circ_0003611 reduces expressions of MYC target genes in MSCs.** **A** Mina53 mRNA level in control hBMSCs and hBMSCs with hsa_circ_0003611 stably silenced. **B** DKC1 mRNA level in control hBMSCs and hBMSCs with hsa_circ_0003611 stably silenced. sh-hsa_circ_0003611: hBMSCs with hsa_circ_0003611 stably silenced. *P<0.05, **P<0.01.
